# Sustained enhancement of photosynthesis in coffee trees grown under free-air CO_2_ enrichment conditions: disentangling the contributions of stomatal, mesophyll, and biochemical limitations

**DOI:** 10.1093/jxb/erv463

**Published:** 2015-10-26

**Authors:** Fábio M. DaMatta, Alice G. Godoy, Paulo E. Menezes-Silva, Samuel C.V. Martins, Lílian M.V.P. Sanglard, Leandro E. Morais, André Torre-Neto, Raquel Ghini

**Affiliations:** ^1^Departamento de Biologia Vegetal, Universidade Federal de Viçosa, 36570-900 Viçosa, MG, Brazil; ^2^Embrapa Instrumentation, Rua Quinze de Novembro, 1452, 13561-206 São Carlos, SP, Brazil; ^3^Embrapa Environment, C.p. 69, 13820-000 Jaguariúna, SP, Brazil

**Keywords:** Carbohydrates, *Coffea arabica* L., FACE, nitrogen, photosynthetic limitations, photosynthetic acclimation, starch.

## Abstract

Coffee (*Coffea* spp.), a globally traded commodity, is a slow-growing tropical tree species that displays an improved photosynthetic performance when grown under elevated atmospheric CO_2_ concentrations ([CO_2_]). To investigate the mechanisms underlying this response, two commercial coffee cultivars (Catuaí and Obatã) were grown using the first free-air CO_2_ enrichment (FACE) facility in Latin America. Measurements were conducted in two contrasting growth seasons, which were characterized by the high (February) and low (August) sink demand. Elevated [CO_2_] led to increases in net photosynthetic rates (*A*) in parallel with decreased photorespiration rates, with no photochemical limitations to *A*. The stimulation of *A* by elevated CO_2_ supply was more prominent in August (56% on average) than in February (40% on average). Overall, the stomatal and mesophyll conductances, as well as the leaf nitrogen and phosphorus concentrations, were unresponsive to the treatments. Photosynthesis was strongly limited by diffusional constraints, particularly at the stomata level, and this pattern was little, if at all, affected by elevated [CO_2_]. Relative to February, starch pools (but not soluble sugars) increased remarkably (>500%) in August, with no detectable alteration in the maximum carboxylation capacity estimated on a chloroplast [CO_2_] basis. Upregulation of *A* by elevated [CO_2_] took place with no signs of photosynthetic downregulation, even during the period of low sink demand, when acclimation would be expected to be greatest.

## Highlight

In free-air CO_2_ enrichment (FACE)-grown coffee trees, elevated [CO_2_] led to sustained increases in photosynthesis, with no change in mesophyll or stomatal conductance and no downregulation of biochemical capacity.

## Introduction

The actual increase in atmospheric CO_2_ concentration ([CO_2_]) is one of the most well-documented aspects of global climatic change. In 2014, [CO_2_] exceeded, for the first time in at least the past 650 000 years, 400 µmol mol^–1^ of air, as recorded at the Mauna Loa Observatory in Hawaii. Indeed, over the last decade, atmospheric [CO_2_] has increased at a rate of approximately 2 µmol mol^–1^ of air year^–1^, and projections suggest that atmospheric [CO_2_] will exceed 936 µmol mol^–1^ of air (Representative Concentration Pathway 8.5) by the end of this century (IPCC, 2013). Given that photosynthesis is limited by the CO_2_ supply at the current atmospheric [CO_2_] (Long *et al.*, 2004), an increase in [CO_2_] is expected to increase the net photosynthetic rate (*A*) of plants (Ainsworth and Long, 2005). In fact, theoretical analyses suggest that *A* values might increase by 38% as atmospheric [CO_2_] increases from 380 to 550 µmol mol^–1^ of air (von Caemmerer and Furbank, 2003). Nevertheless, the results from free-air CO_2_ enrichment (FACE) experiments reveal that the photosynthesis of crop plants fails to match the theoretical increase that could be obtained under elevated [CO_2_] and that increases in *A* do not always translate into concordant increases in biomass production and crop yield (Long *et al.*, 2006; Leakey *et al.*, 2009). In any case, increased *A* under elevated [CO_2_] is accompanied by consistent, although not universal, reductions in stomatal conductance (*g*_s_) (Medlyn *et al.*, 2001). Meta-analyses of FACE experiments have reported mean reductions in *g*_s_ of 16–19% (Ainsworth and Long, 2005; Ainsworth and Rogers, 2007). The dichotomous responses of *A* and *g*_s_ to elevated atmospheric [CO_2_] ultimately lead to improvements in the water-use efficiency of the plant, reduced soil moisture depletion, and ameliorated stress during periods of drought (DaMatta *et al.*, 2010a).

In addition to *g*_s_, an increasing body of evidence suggests that the mesophyll conductance (*g*_m_), which is defined as the conductance for the transfer of CO_2_ from the intercellular airspaces (*C*_i_) to the sites of carboxylation in the chloroplastic stroma (*C*_c_), may also be a key constraint to photosynthesis (Flexas *et al.*, 2012; Sun *et al.*, 2014). However, the potential responses of *g*_m_ to climate changes, including elevated atmospheric [CO_2_], have largely been neglected up to now (Flexas *et al*., 2012, 2014). In contrast to the well-known overall pattern of decreased *g*_s_ under long-term [CO_2_] exposure, the available information shows no general trend for *g*_m_ in plants grown under elevated [CO_2_] (i.e. 500–600 µmol mol^–1^ of air); indeed, no change, either decreased or increased *g*_m_, has been reported, possibly depending on the species and time (Flexas *et al.*, 2012, and references therein). In any case, not considering *g*_m_ in photosynthesis calculations can significantly alter our interpretation of the effects of environmental factors, including CO_2_ supply, on photosynthesis (Sun *et al.*, 2014). For example, mis-interpretations of photosynthetic downregulation (see below) based on decreases in the maximum apparent carboxylation capacity (*V*_cmax_) on a *C*_i_ basis may occur when a decreased *g*_m_ under elevated [CO_2_] is not considered.

In some FACE studies that were conducted with temperate tree species, the initial stimulation of *A* was preserved during the long-term exposure to elevated [CO_2_] (Liberloo etal., 2007; Bader etal., 2010; Streit *et al.*, 2014). Frequently, however, plants that were exposed to elevated [CO_2_] in FACE trials show reductions in photosynthetic capacity (Rogers and Ellsworth, 2002; Hyvönen *et al.*, 2007; Norby etal., 2010), termed photosynthetic downregulation (acclimation), associated with end-product accumulation (Ainsworth and Rogers, 2007; Leakey *et al.*, 2009) that is usually linked to nutrient limitations, particularly nitrogen (N) (Ainsworth and Long, 2005). A reduced or acclimated stimulation of photosynthesis has been mechanistically and quantitatively attributed to decreased *V*_cmax_ and investment in ribulose-1,5-bisphosphate carboxylase/oxygenase (Rubisco) (Ainsworth and Long, 2005), but may also be linked to a reduced ribulose-1,5-bisphosphate (RuBP) regeneration, decreasing the *in vivo* maximum rate of carboxylation that is limited by electron transport (*J*_max_) due to either a lowered electron transport capacity or inorganic phosphate availability in the chloroplast for ATP synthesis (Ainsworth and Rogers, 2007; Kirschbaum, 2011; Zhu *et al.*, 2012). As a rule, limited sink strength predisposes plants to a greater acclimation of photosynthetic capacity and decreases the stimulation of photosynthesis by growth under elevated [CO_2_] (Long *et al.*, 2004; Ainsworth and Rogers, 2007). Overall, trees, particularly fast-growing individuals, display a large sink capacity (root–trunk system) compared with that of annuals, which, to a great extent, explains their higher stimulation of photosynthesis when grown under elevated atmospheric [CO_2_] compared with shrubs and annual crops (Ainsworth and Long, 2005).

Coffee is one of the most heavily globally traded commodities, with retail sales worldwide estimated at US$90 billion (DaMatta *et al.*, 2010b). The coffee tree is a slow-growing evergreen species that displays relatively low *A*, with maximum values typically around or below 10 μmol m^–2^ s^–1^ with current atmospheric [CO_2_] and saturating light (DaMatta, 2004). Such low values have largely been associated with diffusive rather than biochemical limitations to photosynthesis (Batista *et al.*, 2012), particularly diffusional constraints at the stomatal level (Martins *et al.*, 2014). Consequently, coffee trees are expected to largely benefit, in terms of photosynthetic performance, by the elevated atmospheric [CO_2_], considering that increasing atmospheric [CO_2_] increases the gradient that ensures an adequate diffusion of CO_2_ from the atmosphere to the chloroplasts. Indeed, the recent efforts of Ramalho *et al.* (2013), who studied potted coffee plants growing in an enclosure system under different [CO_2_] over 1 year, revealed greater *A* (ranging from 34 to 49%) under elevated (700 μmol CO_2_ mol^–1^ of air) than under normal (380 µmol CO_2_ mol^–1^ of air) [CO_2_]. This positive effect was later confirmed by Ghini *et al.* (2015) in coffee plants that were grown using the first FACE facility in Latin America (ClimapestFACE). Interestingly, *g*_s_ responds little, if at all, to elevated [CO_2_] in coffee; taken together, these results imply that substantial increases in water-use efficiency occur in coffee grown under elevated [CO_2_] conditions (Ramalho *et al.*, 2013; Ghini *et al.*, 2015).

In this study, two commercial coffee cultivars with contrasting crop yields were grown under current ambient and elevated [CO_2_] at the ClimapestFACE facility (Ghini *et al.*, 2015). We demonstrated previously that, integrated over the course of the day, the *A* values that were measured *in situ* were ≥40% higher for the plants that were grown under elevated atmospheric [CO_2_], which may in part explain the enhanced crop yields under elevated [CO_2_] (Ghini *et al.*, 2015). Given these previous results and those of enclosure studies (Ramalho *et al.*, 2013), our main goal was to examine the mechanisms underlying this response. To achieve this goal, we conducted an in-depth analysis of the photosynthetic performance by calculating *g*_m_ to properly parameterize the responses of *A* to *C*_c_ in addition to disentangling the relative contributions of the stomatal, mesophyll, photochemical, and biochemical limitations of photosynthesis. This analysis was conducted in two contrasting growth seasons, which were characterized by strong and weak sink demand, when photosynthetic downregulation, if occurring, would be expected to be minimal and maximal, respectively.

## Materials and methods

### Site description, CO_2_ treatments, plant materials, and samplings

We carried out the experiment using the ClimapestFACE facility located in Jaguariúna (22°43'S, 47°01'W, 570 m above sea level), south-eastern Brazil. The soil at the experimental area is a typical dystroferric red latosol. The climate is humid subtropical, a Cfa type according to the Köppen classification, with hot rainy summers and cold dry winters. Mean monthly air temperature and precipitation were recorded during the experiment (see Supplementary Fig. S1 at *JBX* online).

To mimic coffee agrosystems, the FACE system increased the ambient [CO_2_] in six 10 m diameter ring plots (elevated CO_2_) within a continuous 7 ha coffee field. Six additional 10 m diameter ring plots served as controls, i.e. were left under ambient [CO_2_]. Elevated- and ambient-CO_2_ plots were at least 70 m apart to minimize cross-plot contamination.

Fumigation with CO_2_ began on 25 August 2011. The average atmospheric [CO_2_] at the beginning of the experiment was approximately 390 µmol mol^–1^. The performance of the FACE system was adjusted so that the [CO_2_] as measured at the centre of the ring achieved target levels of 550 µmol mol^–1^ of air. The plots were not enriched with CO_2_ at night. Further details regarding the experimental site set-up and CO_2_ control performance can be found in Ghini *et al.* (2015).

Two coffee (*Coffea arabica* L.) cultivars, cv. Catuaí Vermelho IAC 144 and cv. Obatã IAC 1669-20, were assessed. The latter displays significantly greater (>30%) crop yields than the former, as found in test trials under common agronomic practices. Plantlets with three to four pairs of leaves were transplanted into the plots in March 2011. The cultivars were interspersed in rows that were 1.75 m apart, with 0.60 m between plants in the rows. The plots were located in a continuous 7 ha coffee field of cv. Catuaí Vermelho IAC 144, into which plantlets were transplanted on March 2010; the rows were 3.5 m apart, with 0.60 m between plants in the rows. Fertilization per hectare at planting was accomplished with 2300kg of single super phosphate, 2300kg of dolomitic limestone, 285kg of chloride potassium and 570kg of Yoorin Master 2S^®^ (a granulated fertilizer with fast and slow release of P, Ca, Mg and S). The plants were submitted to routine agricultural practices for commercial coffee bean production, including several applications of herbicides, fungicides, and insecticides. Each tree was fertilized annually with 46g of N, 9g of P and 23g of K plus micronutrients. The fertilizer was applied three times during the growing season, coinciding approximately with periods of supplemental fertilization for most commercial coffee crops in Brazilian Arabica coffee-producing regions. The crop was grown without supplemental irrigation.

Sampling and measurements were carried out in two contrasting periods of the coffee growth cycle: February and August (2013). In February, the vegetative growth rates are relatively low due to competition with reproductive growth (bean-filling phase), and thus assimilate demand is at its maximum; in August, the plants are fruitless (crop harvests are usually completed in June–July), and vegetative growth is negligible. Thus, assimilate demand by the sinks is at its minimum (Silva *et al.*, 2004).

### Gas-exchange and chlorophyll *a* fluorescence measurements

The net rate of carbon assimilation (*A*), stomatal conductance [which was subsequently converted into stomatal conductance to CO_2_ (*g*_s_)] and internal CO_2_ concentration (*C*_i_) were measured simultaneously with chlorophyll *a* fluorescence parameters in the youngest fully expanded leaves [the third or fourth leaf pair from the apex of the plagiotropic (lateral) branches in the upper third of the plants] using an open system under ambient temperature and a [CO_2_] of 390 or 550 µmol mol^–1^ of air, depending on the treatment. All of the measurements, which were conducted using three cross-calibrated infrared gas analysers (LI-6400XT; Li-Cor, Lincoln, NE, USA) using an integrated fluorescence chamber head (LI-6400-40; Li-Cor), were made on clear-sky days during four time periods: 08:00–09:00, 10:00–11:00, 13:00–14:00, and 16:00–17:00 (solar time). To improve the uniformity over the course of the day, the measurements were conducted at the leaf level at an artificial photosynthetically active radiation (PAR) level of 1000 µmol of photons m^–2^ s^–1^. This PAR intensity is sufficiently high to saturate the photosynthetic machinery without causing photoinhibition (Cavatte *et al.*, 2012); in addition, these PAR values approximated the ambient irradiance that was intercepted by the sampled leaves (in their natural angles) in most measurements at each time point. After the leaf tissue was clamped in the leaf chamber, the gas-exchange rates usually stabilized within approximately 3min. In both February and August 2013, the measurements were repeated once using different plants within a given ring of the FACE. Even without supplemental irrigation, the pre-dawn xylem water potential, as measured with a Scholander chamber, was greater than –0.1MPa in both periods, suggesting that our gas-exchange data were not constrained by the water supply.

In light-adapted leaves, the steady-state fluorescence yield (*F*_s_) was measured after registering the gas-exchange parameters. A saturating white light pulse (8000 μmol m^–2^ s^–1^; 0.8 s) was applied to achieve the light-adapted maximum fluorescence (*F*_m_'). The actinic light was then turned off, and far-red illumination was applied (2 μmol m^–2^ s^–1^) to measure the light-adapted initial fluorescence (*F*_0_'). Using the values of these parameters, the photochemical quenching coefficient (*q*_P_) and the capture efficiency of excitation energy by open photosystem (PS) II reaction centres (*F*_v_'/*F*_m_') were estimated (Logan *et al.*, 2007). The actual PSII photochemical efficiency (φ_PSII_) was determined as φ_PSII_=(*F*_m_' – *F*_*s*_)/*F*_m_' following the procedures of Genty *et al.* (1989). The electron transport rate (ETR) was then calculated from the equation ETR*=*φ_PSII×_β×α×photosynthetic photon flux density (PPFD), where α is leaf absorptance, and β reflects the partitioning of absorbed quanta between PSII and PSI (Genty *et al.*, 1989). The product of β and α was determined according to Valentini *et al.* (1995) from the relationship between φ_PSII_ and φ_CO2_ as obtained by varying the light intensity under non-photorespiratory conditions.

The rate of mitochondrial respiration in darkness (*R*_D_) was measured early in the morning in dark-adapted leaves and used to estimate mitochondrial respiration in the light (*R*_L_) according to Lloyd *et al.* (1995) as *R*_L_=[0.5 – 0.05ln(PPFD)]×*R*_D_.

The photorespiratory rate of Rubisco (*R*_P_) was calculated as *R*_P_=1/12[ETR – 4(*A*+*R*_L_)] according to Valentini *et al.* (1995). The *R*_P_/*A*_gross_ ratio was obtained throughout the day by computing the values of *R*_P_, *A*, and *R*_L_. The values of *R*_L_ were corrected for leaf temperature (assessed with temperature sensors coupled to infrared gas analysers over the course of the gas-exchange measurements) using the temperature response for *R*_L_ as described by Bernacchi *et al.* (2001).

Four to six *A*/*C*_i_ curves were obtained *in situ* (from approximately 07:00 to 10:30 in February, and from approximately 08:30 to 12:00 in August, when *g*_s_ values were relatively elevated) from different plants per treatment. These curves were initiated at an ambient [CO_2_] (*C*_a_) of 400 μmol mol^–1^ under a saturating PPFD of 1000 μmol m^–2^ s^–1^. Once a steady state was reached, *C*_a_ was gradually decreased to 50 μmol mol^–1^ of air. Upon the completion of the measurements at low *C*_a_, *C*_a_ was returned to 400 μmol mol^–1^ of air to restore the original *A*. Next, *C*_a_ was increased stepwise to 1600 μmol mol^–1^ of air. The *A*/*C*_i_ curves consisted of 13 different *C*_a_ values. This procedure was successfully applied to the measurements that were conducted in February. In August, however, due to the intrinsically low *g*_s_ for the season (coupled to the negative *g*_s_ response to increases in *C*_a_), we were unable to reliably estimate *C*_i_ at *C*_a_ values greater than 600 μmol mol^–1^. Therefore, *A*/*C*_i_ curves only consisted of seven different *C*_a_ values (<600 μmol mol^–1^). Regardless of the season, corrections for the leakage of CO_2_ into and out of the leaf chamber of the LI-6400 were applied to all gas-exchange data as described by Rodeghiero *et al.* (2007).

### Estimations of the mesophyll conductance to CO_2_ (*g*_m_), the maximum rate of carboxylation (*V*_cmax_), the maximum rate of carboxylation limited by electron transport (*J*_max_), and the chloroplastic [CO_2_] of transition (*C*_c_trans_)

*C*_c_ was estimated according to Harley *et al.* (1992) as follows:

Cc= {Γ*[ETR+8(A+RL)]/[ETR−4(A+RL)]}

where ETR and *A* were obtained from the gas-exchange and chlorophyll *a* fluorescence measurements as conducted under saturating light; *R*_L_ was estimated as described above; and Γ* is the CO_2_ compensation point in the absence of mitochondrial respiration (the conservative value of Γ* for coffee was obtained from Martins *et al.*, 2013). We next estimated *g*_m_ as the slope of the *A* versus *C*_i_ – *C*_c_ relationship as *A*=*g*_m_(*C*_i_ – *C*_c_) so the estimated *g*_m_ was an averaged value over the points used in the relationship (*C*_i_ <350 μmol mol^–1^ of air).

Because all of the available methods for estimating *g*_m_ rely on models that include several assumptions as well as technical limitations and sources of error that need to be considered to obtain reliable estimates of this parameter (Pons *et al.*, 2009), *g*_m_ was also estimated using an alternative approach: the exhaustive dual optimization (EDO) curve-fitting technique of Gu *et al.* (2010). For this purpose, the data from the *A*/*C*_i_ curves were uploaded to the Oak Ridge National Laboratory (USA) website (http://leafweb.ornl.gov), which uses EDO to parameterize *A*/*C*_i_ curves based on the Farquhar–von Caemmerer–Berry model (Gu *et al.*, 2010).

The *g*_m_ values were used to convert *A* – *C*_i_ into *A* – *C*_c_ curves. From these curves, *V*_cmax_ and *J*_max_ were calculated by fitting the mechanistic model of CO_2_ assimilation as proposed by Farquhar *et al.* (1980) using the *C*_c_-based temperature dependence of the kinetic parameters of Rubisco (Bernacchi *et al.*, 2002). The curve-fitting procedures have been detailed elsewhere (Martins *et al.*, 2014). Afterwards, the photosynthetic parameters *V*_cmax_, *J*_max_, and *g*_m_ were normalized to 25 °C using the temperature response equations from Sharkey *et al.* (2007).

The chloroplastic [CO_2_] of transition (*C*_c_trans_), which denotes the transition between the Rubisco- and RuBP regeneration-limited states, was estimated as described by Gu *et al.* (2010):

$$C_{\text{c}\_\text{trans}}=\left(J_{\text{max}}K_{\text{m}}-\text{8}V_{\text{cmax}}\Gamma^{*}\right)/\left(\text{4}V_{\text{cmax}}-J_{\text{max}}\right)$$

where *K*_m_, the effective Michaelis–Menten constant for CO_2_ that considers the competitive inhibition by O_2_, was taken from Martins *et al.* (2013). *C*_c_trans_ was calculated using *V*_cmax_, *J*_max_, *K*_m_, and Г* at the ambient temperatures realized during the gas-exchange measurements at 08:00–09:00h in order to allow proper comparisons with the measured *C*_c_ at this time.

### Quantitative analysis of the limitations of photosynthesis

The overall photosynthetic limitations were partitioned into their functional components [stomatal (*l*_s_), mesophyll (*l*_m_), and biochemical (*l*_b_) limitations] using the values of *g*_s_, *g*_m_, *V*_cmax_, Г*, *K*_m_, and *C*_c_ following the approach that was proposed by Grassi and Magnani (2005) as follows:

$$l_{\text{s}}=\left(g_{\text{tot}}/g_{\text{s}}*\partial A/\partial C_{\text{c}}\right)/\left(g_{\text{tot}}+\partial A/\partial C_{\text{c}}\right)$$

$$l_{\text{m}}=\left(g_{\text{tot}}/g_{\text{m}}*\partial A/\partial C_{\text{c}}\right)/\left(g_{\text{tot}}+\partial A/\partial C_{\text{c}}\right)$$

$$l_{\text{b}}=g_{\text{tot}}/\left(g_{\text{tot}}+\partial A/\partial C_{\text{c}}\right)$$

where *g*_s_ is the stomatal conductance to CO_2_ and *g*_m_ is the mesophyll conductance to CO_2_ according to Harley *et al.* (1992), and *g*_tot_ is the total conductance to CO_2_ from ambient air to chloroplasts (*g*_tot_=1/[(1/*g*_s_)+(1/*g*_m_)]). ∂*A*/∂*C*_c_ was calculated as

$$\partial A/\partial C_{\text{c}}=\left[V_{\text{cmax}}\left(\Gamma^{*}+K_{\text{m}}\right)\right]/{\left(C_{\text{c}}+K_{\text{m}}\right)}^{\text{2}}$$

### Biochemical, N and P, analyses

Leaf discs were collected on clear-sky days prior to flash freezing in liquid nitrogen and subsequent storage at –80 ºC until analysis. For carbohydrate analyses, a 10mg sample of ground tissue was added to pure methanol and the mixture was incubated at 70 °C for 30min. After centrifugation (13 000*g*, 5min), the hexoses (glucose and fructose) and sucrose in the supernatant were quantified; the concentration of starch was determined from the methanol-insoluble pellet as detailed previously (Praxedes *et al.*, 2006; Ronchi *et al.*, 2006). The levels of malate and fumarate were determined exactly as reported elsewhere (Nunes-Nesi *et al.*, 2007).

Leaf samples were used to measure the N (using an elemental analyser; Carlo Erba, Milan, Italy) and P (using routine spectrophotometric methods) contents.

### Statistical analyses

The experiment was designed as a split-plot design with CO_2_ level as the whole-plot factor and cultivar as the subplot factor with six replicates per treatment (ambient and elevated [CO_2_]), and the data were subjected to ANOVA. The treatment differences were also tested using ANOVA. Throughout the text, mean differences were considered significant at *P*≤ 0.05 (see Supplementary Table S1 at *JBX* online).

## Results

Most of the significant treatment effects on the variables analysed were observed for the [CO_2_] factor rather than for the cultivar factor; overall, the traits showed no significant responses to the [CO_2_]×cultivar interaction (see Supplementary Table S1).

**Fig. 1.**
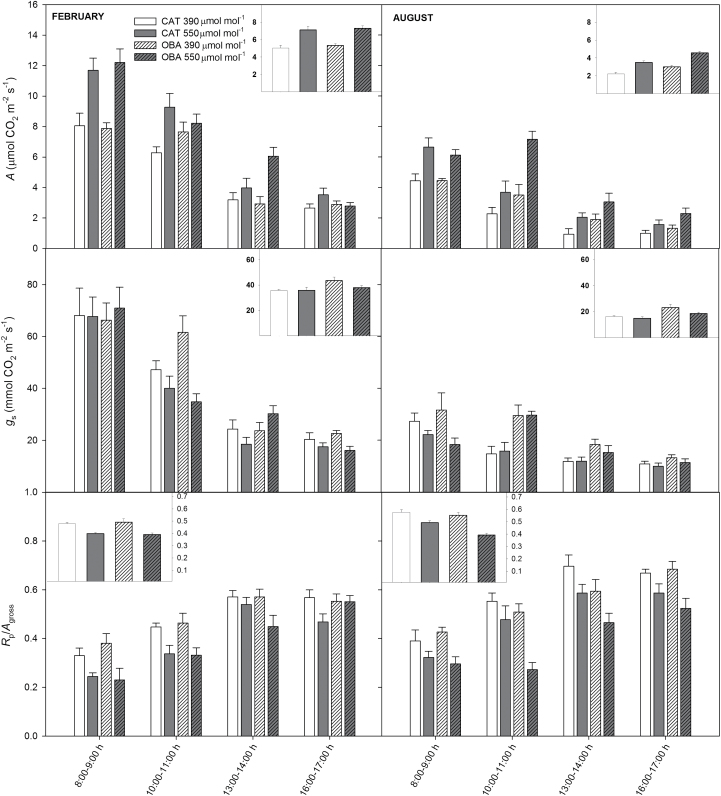
Effect of elevated (550 µmol mol^–1^) and ambient (390 µmol mol^–1^) [CO_2_] on the leaf gas exchange of two coffee cultivars (Catuaí and Obatã) growing in a FACE trial during the growing season (February) and winter (August): net CO_2_ assimilation rate (*A*), stomatal conductance to CO_2_ (*g*_s_), and ratio of photorespiration-to-gross photosynthetic rate (*R*_P_/*A*_gross_). In the insets, the mean diurnal values of *A*, *g*_s_, and *R*_P_/*A*_gross_ are shown. The [CO_2_] used during the gas-exchange measurements was identical to the growth [CO_2_] (390 or 550 µmol mol^–1^). The data for *A* were taken from Ghini *et al.* (2015). *n*=6±SE.

During the growing season, *A* was significantly greater under elevated than under ambient [CO_2_] at two of the four time points in both cultivars (Fig. 1). *g*_s_ did not respond to the CO_2_ enrichment in cv. Catuaí; in cv. Obatã, the *g*_s_ values at two time points were lower under elevated than under ambient [CO_2_] (Fig. 1). The *R*_P_/*A*_gross_ ratio was clearly lower in the morning in both cultivars that were treated with CO_2_, with a less clear pattern in the afternoon (Fig. 1). Notably, diurnally integrated *A* was 40% greater and the *R*_P_/*A*_gross_ ratio was 20% lower under elevated than under ambient [CO_2_], with no significant change in *g*_s_ in either cultivar (Fig. 1). During the period of limited growth (winter), both *A* and *g*_s_ values were, as expected, much lower than during the growing season, whereas an opposite pattern was noted for the *R*_P_/*A*_gross_ ratio (Fig. 1). In all of the winter measurements, both of the cultivars responded to CO_2_ fertilization by increasing *A* significantly, with little, if any, alteration in *g*_s_. The *R*_P_/*A*_gross_ ratio was consistently lower in cv. Obatã under elevated than under ambient [CO_2_], whereas in cv. Catuaí, this ratio was unresponsive to the CO_2_ supply. The diurnally mean *A* values in winter averaged 56% higher in the plants that were treated with CO_2_ than in their untreated counterparts. In cv. Obatã, *g*_s_ values integrated over the course of the day were significantly lower under elevated [CO_2_] (Fig. 1).

Overall, diurnally integrated gas exchange was essentially similar in both cultivars during the growing season (Fig. 1). In winter, *A* was significantly greater in cv. Obatã (irrespective of CO_2_ fertilization), and *R*_P_/*A*_gross_ was lower in this cultivar (only at elevated [CO_2_]) than in cv. Catuaí (Fig. 1).

**Fig. 2.**
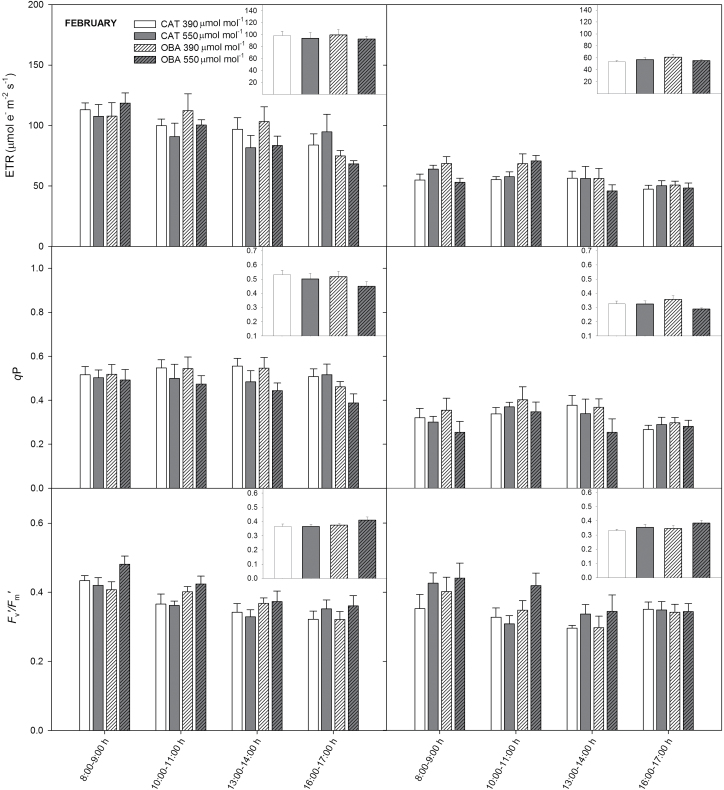
Effect of elevated (550 µmol mol^–1^) and ambient (390 µmol mol^–1^) [CO_2_] on the photochemical parameters of two coffee cultivars (Catuaí and Obatã) growing in a FACE trial during the growing season (February) and winter (August): electron transport rate (ETR), photochemical quenching coefficient (*q*_P_), and capture efficiency of excitation energy by open PSII reaction centres (*F*_v_'/*F*_m_'). In the insets, the mean diurnal values of ETR, *q*_P_, and *F*_v_'/*F*_m_' are shown. The [CO_2_] used during the chlorophyll *a* fluorescence measurements was identical to the growth [CO_2_]. *n*=6±SE.

From the chlorophyll *a* fluorescence analysis, we estimated the ETR, *q*_P_, and *F*_v_'/*F*_m_' over the course of the day (Fig. 2). In both the growing season and winter, all of these traits were virtually unaltered by the CO_2_ treatments. However, we noted that, irrespective of cultivar, the overall values of ETR and *q*_P_ were higher in the growing season than in the phase of restrained growth, whereas *F*_v_'/*F*_m_' varied little, if at all, between seasons.

We analysed *g*_m_ using two independent methods. Overall, similar values and trends for *g*_m_ were observed regardless of the experimental approach that was used to estimate *g*_m_ (Table 1). Therefore, only the *g*_m_ values that were obtained using the method of Harley *et al.* (1992) are discussed below and were used to parameterize the responses of *A* to *C*_c_. In both the growing season and winter, *g*_m_ did not respond to the CO_2_ treatments (Table 1). Both *V*_cmax_ and *J*_max_, estimated on a *C*_c_ and *C*_i_ basis, were unresponsive to the CO_2_ supply in the growing season, as was *V*_cmax_ during the winter (Table 1 and Supplementary Table S2 at *JBX* online). In fact, the absolute *V*_cmax_ values on a *C*_i_ basis were essentially similar regardless of the season (57 μmol CO_2_ m^–2^ s^–1^ on average), as can be deduced from Supplementary Table S2. Notably, had the results been calculated only on a *C*_i_ basis, *V*_cmax_ would be underestimated by approximately 40%, thus highlighting the importance of estimating *g*_m_ to properly describe the responses of *A* to the CO_2_ supply.

**Table 1.** Effect of elevated (550 µmol mol^–1^) and ambient (390 µmol mol^–1^) [CO_2_] on some photosynthetic parameters of two coffee cultivars (Catuaí and Obatã) growing in a FACE trial during the growing season (February) and winter (August) The following values were determined: mesophyll conductance (*g*_m_), estimated using two independent methods: the EDO curve-fitting technique and the method that was proposed by Harley *et al.* (1992); the maximum apparent carboxylation capacity (*V*_cmax_) and the *in vivo* maximum rate of carboxylation as limited by electron transport (*J*_max_), both on a chloroplast [CO_2_] basis; and the chloroplastic [CO_2_] (*C*_c_) and *C*_c_ of transition (*C*_c_trans_). Data for *J*_max_ and *C*_c_trans_ were not obtained in August. *V*_cmax_, *J*_max_ and *g*_m_ were normalized to 25 °C using the temperature response equations from Sharkey *et al.* (2007). *n*=5–6±SE.

Parameter	Catuaí			
	February		August	
	390 µmol mol^–1^	550 µmol mol^–1^	390 µmol mol^–1^	550 µmol mol^–1^
*g*_m_EDO_	0.067±0.012	0.102±0.015	0.072±0.012	0.072±0.015
*g*_m_Harley_	0.073±0.008	0.061±0.007	0.071±0.011	0.105±0.024
*V*_cmax_	61.0±1.8	54.8±2.8	57.3±5.4	58.3±3.3
*J*_max_	95.6±3.9	81.1±4.6	-	-
*C*_c_	152±15	205±15	107±9	182±19
*C*_c_trans_	268±15	231±13	-	-
	Obatã			
	February		August	
	390 µmol mol^–1^	550 µmol mol^–1^	390 µmol mol^–1^	550 µmol mol^–1^
*g*_m_EDO_	0.060±0.015	0.083±0.017	0.088±0.021	0.063±0.010
*g*_m_Harley_	0.091±0.018	0.065±0.007	0.058±0.010	0.082±0.014
*V*_cmax_	55.2±3.3	54.8±2.7	59.9±5.6	52.3±1.9
*J*_max_	86.8±6.2	80.0±5.3	–	–
*C*_c_	173±15	253±31	104±19	165±23
*C*_c_trans_	303±10	231±9	–	–

We also determined data for *C*_c_ and *C*_c_trans_ (Table 1) as estimated in the early morning (08:00–09:00h) when *A* is at its maximum. Overall, *C*_c_ was significantly higher under elevated than ambient [CO_2_], and *C*_c_trans_ (estimated only in the growing season due to the available *J*_max_ data) decreased under elevated [CO_2_]. Regardless of cultivars, *C*_c_ was lower than *C*_c_trans_ under ambient [CO_2_]. Under elevated [CO_2_], the values of *C*_c_ did not differ significantly from those of *C*_c_trans_ irrespective of cultivar (*P*>0.05).

**Fig. 3.**
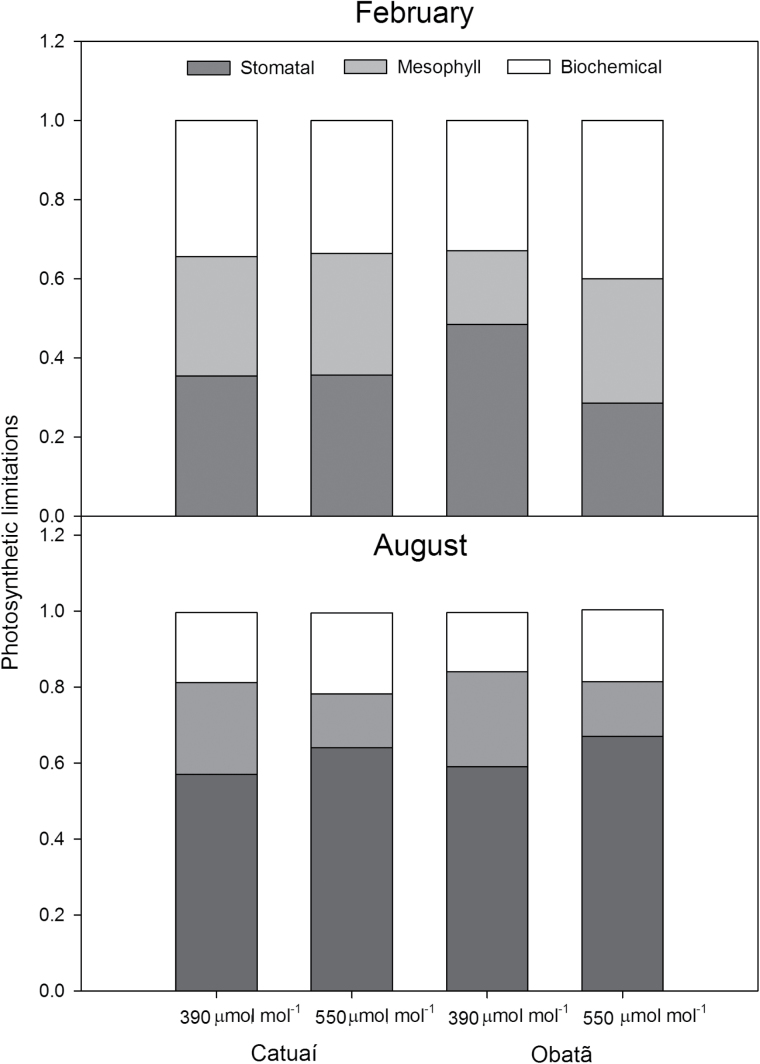
Effect of elevated (550 µmol mol^–1^) and ambient (390 µmol mol^–1^) [CO_2_] on the overall limitations to photosynthesis of two coffee cultivars (Catuaí and Obatã) growing in a FACE trial during the growing season (February) and winter (August): stomatal (*l*_s_), mesophyll (*l*_m_) and biochemical (*l*_b_) limitations. The required gas-exchange parameters used in the estimations were obtained at an ambient [CO_2_] identical to the growth [CO_2_] (390 or 550 µmol mol^–1^). *n*=5–6±SE.

The functional components (*l*_s_, *l*_m_, and *l*_b_) of the overall photosynthetic limitations did not respond to [CO_2_] in cv. Catuaí in the growing season (Fig. 3). In cv. Obatã, *l*_b_ was unresponsive to the [CO_2_] supply, regardless of the season; however, elevated [CO_2_] decreased *l*_s_ (37%) significantly in parallel with an increase in *l*_m_ (73%) in the growing season. In winter, regardless of cultivar, significant changes were only noticeable in *l*_m_, which decreased by 50% under elevated [CO_2_] compared with under ambient [CO_2_]. Importantly, our results indicate that diffusional limitation (*l*_s_+*l*_m_) accounted for the most prominent constraints to photosynthesis (≥60% in the growing season and ≥80% in winter), and these responses were not altered by the CO_2_ supply in either cultivar (Fig. 3). By comparing the two growth periods, the greater diffusional limitations in winter were chiefly associated with increased *l*_s_ (~60% vs ~37% in the growing season), whereas the magnitude of changes in *l*_m_ was much narrower when comparing both seasons. Increased diffusional constraints in winter resulted in a lower *l*_b_ that was approximately half relative to that obtained in the growing season (Fig. 3). We also assessed intrinsic physiological changes as a function of growth CO_2_ by calculating the limitations using different ambient [CO_2_] (390 µmol mol^–1^ of air for the plants grown at 550 µmol mol^–1^ of air and vice versa); the results were nearly identical to those shown previously (Fig. 3) with the exception that *l*_s_ was higher in cv. Obatã even in the winter season (see Supplementary Fig. S2 at *JBX* online).

**Fig. 4.**
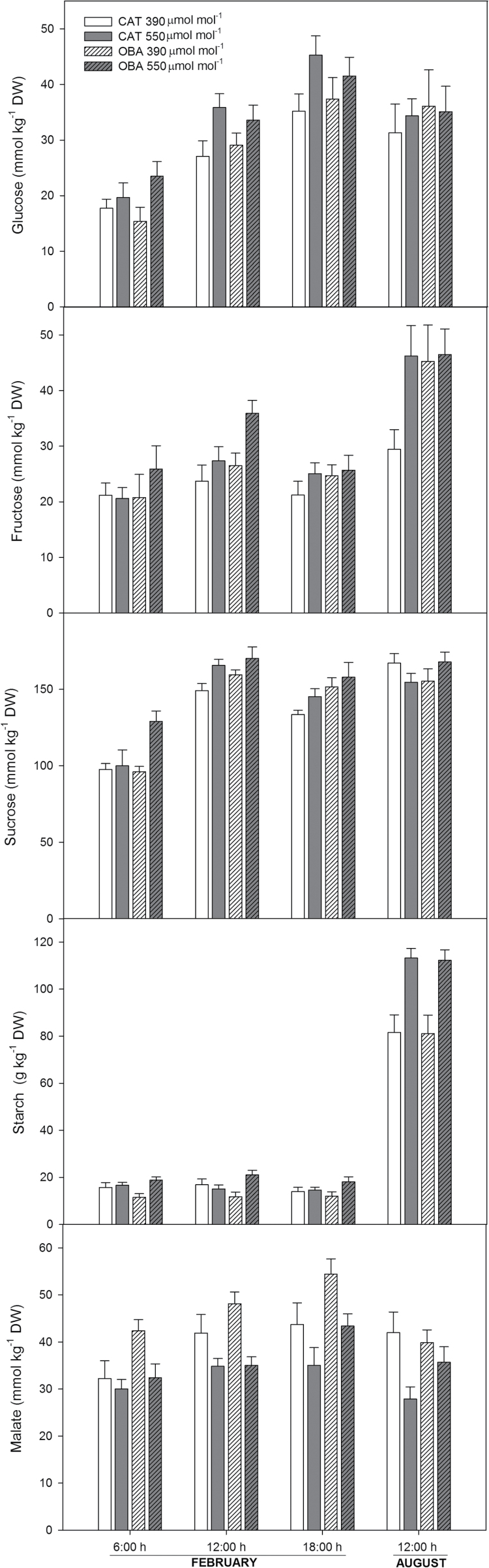
Effect of elevated (550 µmol mol^–1^) and ambient (390 µmol mol^–1^) [CO_2_] on the leaf concentrations of carbohydrates and malate of two coffee cultivars (Catuaí and Obatã) growing in a FACE trial during the growing season (February) and winter (August). *n*=6±SE. DW, dry weight.

In the growing season, the pools of carbohydrates (and malate/fumarate) were measured at the beginning, middle, and end of the photoperiod. Regardless of the cultivar, the concentrations of glucose, fructose, and sucrose were similar or slightly higher in the plants that were treated with CO_2_ compared with their untreated counterparts, but only in a few measurements did these differences reach statistical significance (Fig. 4). The concentrations of starch were not significantly affected by the treatments in cv. Catuaí, whereas in Obatã, the starch levels were consistently higher (64% on average) under elevated than under ambient [CO_2_]. In winter, measurements were conducted only at midday for both cultivars. The glucose and sucrose pools were unaffected by the CO_2_ supply independently of cultivar, whereas the starch pools averaged 38% higher under elevated than under ambient [CO_2_]. Fructose pools increased significantly in response to the CO_2_ supply but only in cv. Catuaí. It should be emphasized that there was an almost invariant total soluble sugar concentration [represented by the sum of glucose, fructose, and sucrose (on average, the total soluble sugars totalled 6.6 and 6.8% of leaf dry weight in the growing season and winter, respectively; data not shown)], whereas the levels of starch were remarkably higher (522% on average) in winter than in the growing season.

We next evaluated the malate and fumarate pools (Fig. 4), given that the concentration of these organic acids often changes when the carbon balance is altered. In the growing season, the levels of malate were not significantly affected by the treatments in cv. Catuaí; in cv. Obatã, malate pools were consistently lower (24% on average) over the course of the day under elevated compared with under ambient [CO_2_]. In winter, malate pools were significantly lower (33%) under elevated than under ambient [CO_2_] in cv. Catuaí, whereas these pools were not affected by the CO_2_ treatment in cv. Obatã. Regardless of the season, fumarate was not detected in this study.

The leaf N and P concentrations were approximately 3.5 and 1.5%, respectively, on a dry-weight basis and remained invariant regardless of treatment (data not shown). These concentrations are within an optimal range for coffee.

## Discussion

In this study, we examined in detail the underlying mechanism associated with the photosynthetic enhancement that we demonstrated previously in coffee trees when grown with supplemental CO_2_ (Ghini *et al.*, 2015). First (and as expected), a higher *A* under elevated [CO_2_] was associated not only with improved carboxylation rates coupled with a higher availability of CO_2_ as substrate (higher *C*_c_) but also with a relatively higher carboxylation over oxygenation activity of Rubisco, resulting in decreased *R*_P_, here noted through the lower *R*_P_/*A*_gross_ ratio under elevated [CO_2_] (Fig. 1).

Given that the stimulation of *A* by CO_2_ fumigation would increase the ATP demand that is required for RuBP regeneration and that the control of photosynthesis shifts from Rubisco-limited to RuBP regeneration-limited (Ainsworth and Rogers, 2007), we next analysed whether any impairment in leaf photochemistry could constrain the maximization of *A* under elevated [CO_2_] (Fig. 2). We found that the CO_2_ stimulation of *A* was not accompanied by concordant alterations in the efficiency of the excitation energy as captured by the open PSII reaction centres (estimated as *F*_v_'/*F*_m_') and in the fraction of absorbed light that is dissipated photochemically (estimated as *q*_P_). Importantly, the ETR values by far exceeded the photochemical needs that were required to support the observed *A* values (Martins *et al.*, 2013), irrespective of treatments. Therefore, our data indicated that photochemical events are unlikely to have limited CO_2_ fixation in this study. In any case, seasonal photochemical adjustments (decreased *q*_P_ and ETR in winter) occurred independently of the CO_2_ supply, but these adjustments should represent a consequence, rather than a cause, of the decreases in *A* in winter. In other words, adjustments probably occurred because the light-capture and light-utilization processes are imbalanced in coffee in winter, and thus adjustments in leaf photochemistry may be a proper way of avoiding the occurrence of photoinhibition in coffee (Chaves *et al.*, 2008; Pompelli *et al.*, 2010), as could also be deduced here by the unchanging variable-to-maximum chlorophyll fluorescence ratio (data not shown).

We subsequently quantified the distribution of the overall photosynthetic limitations between diffusional and biochemical processes. To reach this goal, we first estimated *g*_m_ and found no *g*_m_ acclimation to elevated CO_2_ (Table 1). Regardless of the CO_2_ supply, we demonstrated that the major limitations to photosynthesis in coffee are linked to diffusional constraints (Fig. 3), with greater values of *l*_s_ than those of *l*_m_, as also reported previously for potted coffee seedlings grown under ambient [CO_2_] (Martins *et al.*, 2014). In addition, we found, relative to the growing season, an exacerbation of diffusional constraints to coffee’s photosynthesis in winter that was almost entirely traceable by further increases in *l*_s_. Such an increase may be a consequence of the stomatal sensitivity to low nocturnal temperatures in *C. arabica* (Barros *et al.*, 1997; Silva *et al.*, 2004). In contrast to other studies suggesting that mesophyll constraints to photosynthesis are of a similar magnitude as stomatal limitations (Warren, 2008; Flexas *et al.*, 2012), we found that *l*_s_ was exacerbated in coffee trees in the afternoon, particularly because *g*_s_ peaked in the early morning and decreased sharply in the afternoon, reaching values that were typically below 40 mmol H_2_O m^–2^ s^–1^ (Chaves *et al.*, 2008; DaMatta *et al.*, 2008) as a consequence of increasing vapour pressure deficit. Therefore, as coffee trees display an inherently low *g*_s_ and *g*_m_ (see also Martins *et al.*, 2014), it is expected that this species might benefit remarkably from increasing [CO_2_] relatively more than other plant species with lower diffusional limitations to photosynthesis (Flexas *et al.*, 2014).

Notably, the absence of CO_2_-induced alterations in *l*_b_ is consistent with unaltered parameters related to biochemistry (*J*_max_ and *V*_cmax_ on a *C*_c_ basis) and N and P concentrations, suggesting that the amount of resources (e.g. N) that were allocated to electron carriers and Calvin cycle enzymes remained unchanged. Additionally, unchanged *V*_cmax_ has been associated with unaltered Rubisco amounts and/or activation state. Overall, these results, which are consistent with a lack of photosynthetic acclimation, contradict what has been noted in many studies in which *V*_cmax_, in particular, has been shown to be downregulated in response to elevated [CO_2_] (Ainsworth and Long, 2005, and references therein). Our results, however, agree with the report of Ramalho *et al.* (2013), who suggested a lack of photosynthetic downregulation in coffee seedlings grown in large pots in enclosure systems. Other evidence demonstrates that photosynthetic downregulation was unlikely to have occurred in this study: (i) the photosynthetic enhancement due to elevated [CO_2_] was independent of the CO_2_ supply because the *A* values did not differ significantly between coffee trees that were grown under ambient or elevated [CO_2_] when measurements were conducted at 390 or 550 µmol CO_2_ mol^–1^ of air (Ghini *et al.*, 2015); and (ii) such an enhancement occurred regardless of the higher carbohydrate accumulation under elevated [CO_2_].

In the early morning, when the *A* and *g*_s_ values are at their maxima, the *A* in the plants under elevated [CO_2_] was at the region co-limited by Rubisco and RuBP regeneration, given that *C*_c_ was statistically equal to *C*_c_trans_ regardless of cultivar, as observed in the growing season (Table 1). On the one hand, this response means no investment excess in Rubisco or ETR; on the other hand, it would also imply diminishing returns in *A* with further increments in [CO_2_] and no adjustments in *g*_s_ and *g*_m_ (Martins *et al.*, 2014). Such a fast transition from the Rubisco-limited to the electron transport-limited phase is due to a higher affinity for CO_2_ displayed by coffee’s Rubisco (Martins *et al.*, 2013, 2014), which, despite being highly advantageous under low CO_2_ conditions, diminishes the benefit from elevated [CO_2_] (Martins *et al.*, 2014). In contrast, in winter, the remarkably higher diffusional limitations to *A* coupled with unaltered *V*_cmax_ (assuming a constant *J*_max_/*V*_cmax_ ratio) would lead the *C*_c_ values to be in the Rubisco-limited phase, where the response of *A* to *C*_c_ is more prominent. This pattern, regardless of season, is also expected from midday onwards due to the low *g*_s_ values (Fig. 1) and highlights the importance of elevated [CO_2_] for overcoming the high constraints to CO_2_ diffusion for maximizing *A* in coffee.

The remarkable increases in starch levels with no detectable alteration in *V*_cmax_ in winter suggests, overall, that coffee displays a relatively high ability to accumulate starch in its leaf tissues without compromising its photosynthetic performance. Although there are a number of studies supporting direct couplings between starch accumulation and photosynthetic acclimation in response to elevated CO_2_ (e.g. Eguchi *et al.*, 2008; Dawes *et al.*, 2013), our results agree with other studies in which uncoupling between high starch pools and photosynthetic downregulation has not been observed, as reported for fast-growing poplar clones (grown in FACE under elevated [CO_2_]) displaying a combination of a high capacity for starch synthesis and a high sink demand; Davey *et al.*, 2006; Liberloo *et al.*, 2009). Given that starch concentrations changed paralleling an almost invariant total soluble sugar concentrations (Fig. 4), it is tempting to suggest that increased starch levels, especially under elevated [CO_2_], instead of feeding back to decrease photosynthetic performance, allowed the coffee trees to avoid photosynthetic acclimation by preventing the cycling and/or accumulation of soluble sugars. When it occurs, soluble sugar accumulation could in turn more directly repress photosynthetic gene expression (Paul and Foyer, 2001; Paul and Pellny, 2003) and ultimately provoke downregulation. Nevertheless, in sharp contrast to fast-growing poplar clones, we demonstrated here a lack of photosynthetic downregulation in a tropical, slow-growing species, and this fact was observed not only during the growing season but also during the period of the lowest sink demand when acclimation would be expected. This information implies a sustained ability of coffee trees that are rooted freely in the soil to benefit from elevated [CO_2_]; it additionally highlights the fact that the remarkable decreases in *A* in winter (Fig. 1) could not be associated with a starch build-up and concomitant photosynthetic acclimation but rather are chiefly associated with diffusional constraints regardless of the CO_2_ supply.

In the growing season, there were no cultivar differences in *A*, but cv. Obatã displayed increased starch concentrations, which might support an improved bean-filling capacity; in winter, on the other hand, cv. Obatã displayed significantly higher *A* values than cv. Catuaí with similar leaf carbohydrate pools, which might be translated into a higher photoassimilate production that could be stored in the root–trunk system, guaranteeing improved vegetative growth when the environmental conditions become more conducive for growth. Overall, these facts may help to explain why cv. Obatã is better able to sustain higher vegetative growth rates and crop yields than cv. Catuaí under elevated [CO_2_] (Ghini *et al.*, 2015).

In conclusion, we demonstrated under plantation conditions that coffee photosynthesis is strongly limited by diffusional constraints, particularly at the stomata level, and that this pattern is little, if at all, affected by elevated [CO_2_]. Stimulation of *A* by elevated [CO_2_] occurred without any photosynthetic downregulation, even during the period of lowest sink demand by the crop. Given these facts, we therefore suggest that coffee will greatly benefit from the rise in atmospheric [CO_2_], as will other species where photosynthesis is largely limited by CO_2_ diffusion.

## Supplementary data

Supplementary data are available at *JXB* online.

**Supplementary Fig. S1.** The time course of mean monthly air temperature and precipitation at the experimental site from January 2012 through December 2013.

**Supplementary Fig. S2.** The effect of elevated (550 µmol mol^–1^ of air) or ambient (390 µmol mol^–1^ of air) [CO_2_] on the overall limitations to photosynthesis of two coffee cultivars (Catuaí and Obatã) calculated with different ambient [CO_2_] (390 µmol mol^–1^ of air for the plants grown at 550 µmol mol^–1^ of air and vice versa).

**Supplementary Table S1.** The results of ANOVA for the effects of cultivar (Cult), CO_2_ concentration (CO_2_), and their interaction.

**Supplementary Table S2.** The effect of elevated (550 µmol mol^–1^ of air) or ambient (390 µmol mol^–1^ of air) [CO_2_] on the maximum carboxylation rate of Rubisco (*V*_cmax_) and the *in vivo* maximum rate of carboxylation as limited by electron transport (*J*_max_), both on an intercellular [CO_2_] basis, of two coffee cultivars (Catuaí and Obatã) growing in a FACE trial during the growing season (February) and winter (August).

Supplementary Data

## References

[CIT0001] Ainsworth EA Long SP 2005 What have we learned from 15 years of free air CO_2_ enrichment (FACE)? A meta-analytic review of the responses of photosynthesis, canopy properties and plant production to rising CO_2_ . New Phytologist 165, 351–372. 15720649 10.1111/j.1469-8137.2004.01224.x

[CIT0002] Ainsworth EA Rogers A 2007 The response of photosynthesis and stomatal conductance to rising [CO_2_]: mechanisms and environmental interactions. Plant, Cell and Environment 30, 258–270. 10.1111/j.1365-3040.2007.01641.x 17263773

[CIT0003] Bader MK-F Siegwolf R Körner C 2010 Sustained enhancement of photosynthesis in mature deciduous forest trees after 8 years of free air CO_2_ enrichment. Planta 232, 1115–1125. 20700744 10.1007/s00425-010-1240-8

[CIT0004] Barros RS Mota JWS DaMatta FM Maestri M 1997 Decline of vegetative growth in Coffea arabica L. in relation to leaf temperature, water potential and stomatal conductance. Field Crops Research 54, 65–72.

[CIT0005] Batista KD Araújo WL Antunes WC Cavatte PC Moraes GABK Martins SCV DaMatta FM 2012 Photosynthetic limitations in coffee plants are chiefly governed by diffusive factors. Trees 26, 459–468.

[CIT0006] Bernacchi CJ Portis AR Nakano H von Caemmerer S Long SP 2002 Temperature response of mesophyll conductance. Implications for the determination of Rubisco enzyme kinetics and for limitations to photosynthesis *in vivo* . Plant Physiology 130, 1992–1998. 12481082 10.1104/pp.008250 PMC166710

[CIT0007] Bernacchi CJ Singsaas EL Pimentel C Portis AR Long SP 2001 Improved temperature response functions for models of Rubisco-limited photosynthesis. Plant, Cell and Environment 24, 253–259.

[CIT0008] Cavatte PC Oliveira AAG Morais LE Martins SCV Sanglard LMVP DaMatta FM 2012 Could shading reduce the negative impacts of drought on coffee? A morphophysiological analysis. Physiologia Plantarum 114, 111–122. 21939445 10.1111/j.1399-3054.2011.01525.x

[CIT0009] Chaves ARM Ten-Caten A Pinheiro HA Ribeiro A DaMatta FM 2008 Seasonal changes in photoprotective mechanisms of leaves from shaded and unshaded field-grown coffee (*Coffea arabica* L.). Trees 22, 351–361.

[CIT0010] DaMatta FM 2004 Ecophysiological constraints on the production of shaded and unshaded coffee: a review. Field Crops Research 86, 99–114.

[CIT0011] DaMatta FM Cunha RL Antunes WC Martins SVC Araújo WL Fernie AR Moraes GABK 2008 In field-grown coffee trees source-sink manipulation alters photosynthetic rates, independently of carbon metabolism, via alterations in stomatal function. New Phytologist 178, 348–357. 18266616 10.1111/j.1469-8137.2008.02367.x

[CIT0012] DaMatta FM Grandis A Arenque BC Buckeridge MS 2010 *a* Impacts of climate changes on crop physiology and food quality. Food Research International 43, 1814–1823.

[CIT0013] DaMatta FM Ronchi CP Maestri M Barros RS 2010 *b* Coffee: environment and crop physiology. In: DaMatta FM, ed. Ecophysiology of tropical tree crops. New York: Nova Science Publishers, 181–216.

[CIT0014] Davey PA Olcer H Zakhleniuk O Bernacchi CJ Calfapietra C Long SP Raines CA 2006 Can fast-growing plantation trees escape biochemical down-regulation of photosynthesis when grown throughout their complete production cycle in the open air under elevated carbon dioxide? Plant, Cell and Environment 29, 1235–1244. 10.1111/j.1365-3040.2006.01503.x 17080946

[CIT0015] Dawes MA Hagedorn F Handa IT Streit K Ekblad A Rixen C Körner C Hättenschwiler S 2013 An alpine treeline in a carbon dioxide-rich world: Synthesis of a nine-year free-air carbon dioxide enrichment study. Oecologia 171, 623–637. 23340765 10.1007/s00442-012-2576-5

[CIT0016] Eguchi N Karatsu K Ueda T Funada R Takagi K Hiura T Sasa K Koike T 2008 Photosynthetic responses of birch and alder saplings grown in a free air CO_2_ enrichment system in northern Japan. Trees 22, 437–447.

[CIT0017] Farquhar GD von Caemmerer S Berry JA 1980 A biochemical model of photosynthetic CO_2_ assimilation in leaves of C3 species. Planta 149, 78–90. 24306196 10.1007/BF00386231

[CIT0018] Flexas J Barbour MM Brendel O 2012 Mesophyll diffusion conductance to CO_2_: an unappreciated central player in photosynthesis. Plant Science 193–194, 70–84. 10.1016/j.plantsci.2012.05.009 22794920

[CIT0019] Flexas J Carriquí M Coopman RE Gago J Galmés J Martorell S Morales F Diaz-Espejo A 2014 Stomatal and mesophyll conductances to CO_2_ in different plant groups: underrated factors for predicting leaf photosynthesis responses to climate change? Plant Science 226, 41–48. 25113449 10.1016/j.plantsci.2014.06.011

[CIT0020] Genty B Briantais JM Baker NR 1989 The relationship between the quantum yield of photosynthetic electron-transport and quenching of chlorophyll fluorescence. Biochimica et Biophysica Acta 990, 87–92.

[CIT0021] Ghini R Torre-Neto A Dentzien AFM Gerreiro-Filho O Iost R Patrício FRA Prado JSM Thomaziello RA Bettiol W DaMatta FM 2015 Coffee growth, pest and yield responses to free-air CO_2_ enrichment. Climatic Change 132, 307–320.

[CIT0022] Grassi G Magnani F 2005 Stomatal, mesophyll conductance and biochemical limitations to photosynthesis as affected by drought and leaf ontogeny in ash and oak trees. Plant, Cell and Environment 28, 834–849.

[CIT0023] Gu L Pallardy SG Tu K Law BE Wullschleger SD 2010 Reliable estimation of biochemical parameters from C₃ leaf photosynthesis-intercellular carbon dioxide response curves. Plant, Cell and Environment 33, 1852–1874. 10.1111/j.1365-3040.2010.02192.x 20561254

[CIT0024] Harley PC Loreto F Di Marco G Sharkey TD 1992 Theoretical considerations when estimating the mesophyll conductance to CO_2_ flux by analysis of the response of photosynthesis to CO_2_ . Plant Physiology 98, 1429–1436. 16668811 10.1104/pp.98.4.1429 PMC1080368

[CIT0025] Hyvönen R Ågren GI Linder S 2007 The likely impact of elevated [CO_2_], nitrogen deposition, increased temperature and management on carbon sequestration in temperate and boreal forest ecosystems: a literature review. New Phytologist 173, 463–480. 17244042 10.1111/j.1469-8137.2007.01967.x

[CIT0026] **IPCC**. 2013 Climate change 2013: The physical science basis . Cambridge, UK/New York: Cambridge University Press.

[CIT0027] Kirschbaum MUF 2011 Does enhanced photosynthesis enhance growth? Lessons learned from CO_2_ enrichment studies. Plant Physiology 155, 117–124. 21088226 10.1104/pp.110.166819 PMC3075783

[CIT0028] Leakey ADB Ainsworth EA Bernacchi CJ Alistair R Long SP Ort DR 2009 Elevated CO_2_ effects on plant carbon, nitrogen, and water relations: six important lessons from FACE. Journal of Experimental Botany 60, 2859–2876. 19401412 10.1093/jxb/erp096

[CIT0029] Liberloo M Lukac M Calfapietra C Hoosbeek MR Gielen B Miglietta F. Scarascia-Mugnozza GE Ceulemans R 2009 Coppicing shifts CO_2_ stimulation of poplar productivity to above-ground pools: a synthesis of leaf to stand level results from the POP/EUROFACE experiment. New Phytologist 182, 331–346. 19207687 10.1111/j.1469-8137.2008.02754.x

[CIT0030] Liberloo M Tulva I Raim O Kull O Ceulemans R 2007 Photosynthetic stimulation under long-term CO_2_ enrichment and fertilization is sustained across a closed *Populus* canopy profile (EUROFACE). New Phytologist 173, 537–549. 17244048 10.1111/j.1469-8137.2006.01926.x

[CIT0031] Lloyd J Chin Wong S Styles JM Batten D Priddle R Turnbull C McConchie CA 1995 Measuring and modelling whole-tree gas exchange. Australian Journal of Plant Physiology 22, 987–1000.

[CIT0032] Logan BA Adams WW Demmig-Adams B 2007 Avoiding common pitfalls of chlorophyll fluorescence analysis under field conditions. Functional Plant Biology 34, 853–859. 10.1071/FP07113 32689413

[CIT0033] Long SP Ainsworth EA Leakey ADB Ort D 2006 Food for thought: lower-than-expected crop yield stimulation with rising CO_2_ conditions. Science 312, 1918–1921. 16809532 10.1126/science.1114722

[CIT0034] Long SP Ainsworth EA Rogers A Ort DR 2004 Rising atmospheric carbon dioxide: plants FACE the future. Annual Review of Plant Biology 55, 591–628. 10.1146/annurev.arplant.55.031903.141610 15377233

[CIT0035] Martins SVC Galmés J Cavatte PC Pereira LF Ventrella MC DaMatta FM 2014 Understanding the low photosynthetic rates of sun and shade coffee leaves: Bridging the gap on the relative roles of hydraulic, diffusive and biochemical constraints to photosynthesis. PLoS ONE 9, e95571. 24743509 10.1371/journal.pone.0095571 PMC3990704

[CIT0036] Martins SCV Galmés J Molins A DaMatta FM 2013 Improving the estimation of mesophyll conductance: on the role of electron transport rate correction and respiration. Journal of Experimental Botany 64, 3285–3298. 23833194 10.1093/jxb/ert168 PMC3733151

[CIT0037] Medlyn BE Barton CVM Broadmeadow MSJ 2001 Stomatal conductance of forest species after long-term exposure to elevated CO_2_ concentration: a synthesis. New Phytologist 149, 247–264. 10.1046/j.1469-8137.2001.00028.x 33874628

[CIT0038] Norby RJ Warren JM Iversen CM Medlyn BE McMurtrie RE 2010 CO_2_ enhancement of forest productivity constrained by limited nitrogen availability. Proceedings of the National Academy of Sciences, USA 107, 19368–9373. 10.1073/pnas.1006463107 PMC2984154 20974944

[CIT0039] Nunes-Nesi A Carrari F Gibon Y Sulpice R Lytovchenko A Fisahn J Graham J Ratcliffe RG Sweetlove LJ Fernie AR 2007 Deficiency of mitochondrial fumarase activity in tomato plants impairs photosynthesis via an effect on stomatal function. The Plant Journal 50, 1093–106. 17461782 10.1111/j.1365-313X.2007.03115.x

[CIT0040] Paul MJ Foyer CH 2001 Sink regulation of photosynthesis. Journal of Experimental Botany 52, 1383–1400. 11457898 10.1093/jexbot/52.360.1383

[CIT0041] Paul MJ Pellny TK 2003 Carbon metabolite feedback regulation of leaf photosynthesis and development. Journal of Experimental Botany 54, 539–547. 12508065 10.1093/jxb/erg052

[CIT0042] Pompelli MF Martins SCV Antunes WC Chaves ARM DaMatta FM 2010 Photosynthesis and photoprotection in coffee leaves is affected by nitrogen and light availabilities in winter conditions. Journal of Plant Physiology 167, 1052–1060. 20381192 10.1016/j.jplph.2010.03.001

[CIT0043] Pons TL Flexas J von Caemmerer S Evans JR Genty B Ribas-Carbó M Brugnoli E 2009 Estimating mesophyll conductance to CO_2_: methodology, potential errors and recommendations. Journal of Experimental Botany 60, 2217–2234. 19357431 10.1093/jxb/erp081

[CIT0044] Praxedes SC DaMatta FM Loureiro ME Ferrão MAG Cordeiro AT 2006 Effects of long-term soil drought on photosynthesis and carbohydrate metabolism in mature robusta coffee (*Coffea canephora* Pierre var. *kouillou*) leaves. Environmental and Experimental Botany 56, 263–273.

[CIT0045] Ramalho JC Rodrigues AP Semedo JN 2013 Sustained photosynthetic performance of *Coffea* spp. under long-term enhanced [CO_2_]. PLoS ONE 8, e82712. 24324823 10.1371/journal.pone.0082712 PMC3855777

[CIT0046] Rodeghiero M Niinemets U Cescatti A 2007 Major diffusion leaks of clamp-on leaf cuvettes still unaccounted: how erroneous are the estimates of Farquhar *et al*. model parameters? Plant, Cell and Environment 30, 1006–1022. 10.1111/j.1365-3040.2007.001689.x 17617828

[CIT0047] Rogers A Ellsworth DS 2002 Photosynthetic acclimation of *Pinus taeda* (loblolly pine) to long-term growth in elevated pCO_2_ (FACE). Plant, Cell and Environment 25, 851–858.

[CIT0048] Ronchi CP DaMatta FM Batista KD Moraes GABK Loureiro ME Ducatti C 2006 Growth and photosynthetic down-regulation in *Coffea arabica* in response to restricted root volume. Functional Plant Biology 33, 1013–1023. 10.1071/FP06147 32689312

[CIT0049] Sharkey TD Bernacchi CJ Farquhar GD Singsaas EL 2007 Fitting photosynthetic carbon dioxide response curves for C3 leaves. Plant, Cell and Environment 30, 1035–1040. 10.1111/j.1365-3040.2007.01710.x 17661745

[CIT0050] Silva EA DaMatta FM Ducatti C Regazzi AJ Barros RS 2004 Seasonal changes in vegetative growth and photosynthesis of arabica coffee trees. Field Crops Research 89, 349–357.

[CIT0051] Streit K Siegwolf RTW Hagedorn F Schaub M Buchmann N 2014 Lack of photosynthetic or stomatal regulation after 9 years of elevated [CO_2_] and 4 years of soil warming in two conifer species at the alpine treeline. Plant, Cell and Environment 37, 315–336. 10.1111/pce.12197 24003840

[CIT0052] Sun Y Gu L Dickinson RE 2014 Asymmetrical effects of mesophyll conductance on fundamental photosynthetic parameters and their relationships estimated from leaf gas exchange measurements. Plant, Cell and Environment 37, 978–994. 10.1111/pce.12213 24117476

[CIT0053] Valentini R Epron D Angelis D Matteucci G Dreyer E 1995 *In situ* estimation of net CO_2_ assimilation, photosynthetic electron flow and photorespiration in Turkey oak (*Quercus cerris* L.) leaves: diurnal cycles under different levels of water supply. Plant, Cell and Environment 18, 631–640.

[CIT0054] von Caemmerer S Furbank RT 2003 The C_4_ pathway: an efficient CO_2_ pump. Photosynthesis Research 77, 191–207. 16228376 10.1023/A:1025830019591

[CIT0055] Warren CR 2008 Stand aside stomata, another actor deserves centre stage: the forgotten role of the internal conductance to CO_2_ transfer. Journal of Experimental Botany 59, 1475–1487. 17975206 10.1093/jxb/erm245

[CIT0056] Zhu C Ziska L Zhu J Zeng Q Xie Z Tang H Jia X Hasegawa T 2012 The temporal and species dynamics of photosynthetic acclimation in flag leaves of rice (*Oryza sativa*) and wheat (*Triticum aestivum*) under elevated carbon dioxide. Physiologia Plantarum 145, 395–405. 22268610 10.1111/j.1399-3054.2012.01581.x

