# Supplementary material for: Sustained enhancement of photosynthesis in coffee trees grown under free-air CO2 enrichment conditions: disentangling the contributions of stomatal, mesophyll, and biochemical limitations
Source: J Exp Bot. 2015 Oct 26;67(1):341–52. doi: 10.1093/jxb/erv463 (PMC4682438; doi:10.1093/jxb/erv463)
Supplement: Supplementary Data [file supp_67_1_341__index.html]

Sustained enhancement of photosynthesis in coffee trees grown under free-air CO2 enrichment conditions: disentangling the contributions of stomatal, mesophyll, and biochemical limitations — Sustained enhancement of photosynthesis in coffee trees grown under free-air CO2 enrichment conditions: disentangling the contributions of stomatal, mesophyll, and biochemical limitations — Supplementary Data 

# Sustained enhancement of photosynthesis in coffee trees grown under free-air CO2 enrichment conditions: disentangling the contributions of stomatal, mesophyll, and biochemical limitations

## Supplementary Data

Data files

- Supplementary Data - Supplementary Data
