# Supplementary material for: Sustained enhancement of photosynthesis in coffee trees grown under free-air CO2 enrichment conditions: disentangling the contributions of stomatal, mesophyll, and biochemical limitations
Source: J Exp Bot. 2015 Oct 26;67(1):341–52. doi: 10.1093/jxb/erv463 (PMC4682438; doi:10.1093/jxb/erv463)
Supplement: Supplementary Data [file supp_erv463_Supplementary_material.pdf]

Supplementary Data

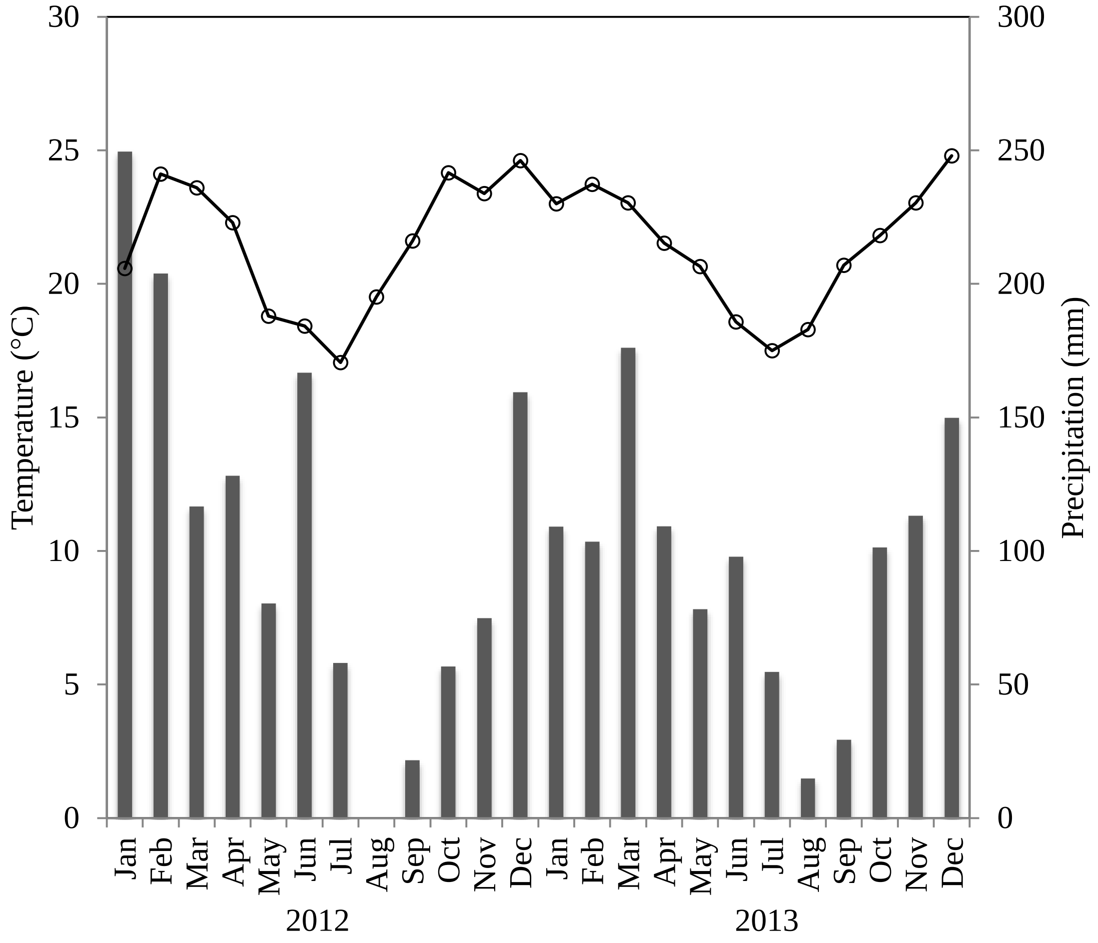

Supplementary Figure S1. The time course of mean monthly air temperature and precipitation at the experimental site from January 2012 through December 2013.

Supplementary Table S1. The results of ANOVA (P-values) for the effects of cultivar (Cult), CO_2 concentration (CO_2) and their interaction are presented for the tested net CO_2 assimilation rate (A), stomatal conductance to CO_2 (g_s), ratio of photorespiration-to-gross photosynthetic rate (R_P/A_{gross}), electron transport rate (ETR), photochemical quenching coefficient (q_P), capture efficiency of excitation energy by open photosystem II reaction centres (F_v'/F_m'), stomatal limitation (l_s), mesophyll limitation (l_m), biochemical limitation (l_b), carbohydrate and malate pools, mesophyll conductance estimated using the Exhaustive Dual Optimization (EDO) curve-fitting technique (g_{m_EDO}) or the method that was proposed by Harley *et al.* (1992) (g_{m_Harley}), the maximum apparent carboxylation capacity (V_{cmax}) and the *in vivo* maximum rate of carboxylation as limited by electron transport (J_{max}) both on a chloroplast [CO_2] (C_c) or an intercellular (C_i) basis, the chloroplastic [CO_2] (C_c), the intercellular [CO_2] (C_i), and C_c or C_i of transition (C_{c_trans} or C_{i_trans}).

Parameter		February			August		
		Cult	CO_2	Cult x CO_2	Cult	CO_2	Cult x CO_2
A	8-9:00 h	0.822	<.0001	0.655	0.553	0.000	0.532
	10-11:00 h	0.804	0.011	0.072	0.001	0.001	0.081
	13-14:00 h	0.104	0.001	0.037	0.026	0.013	0.960
	16-17:00 h	0.435	0.219	0.123	0.065	0.010	0.477
	Diurnal mean	0.452	<.0001	0.865	<.0001	<.0001	0.401
g_s	8-9:00 h	0.929	0.788	0.765	0.944	0.031	0.313
	10-11:00 h	0.325	0.001	0.040	0.006	0.318	0.455
	13-14:00 h	0.084	0.913	0.056	0.090	0.998	0.684
	16-17:00 h	0.816	0.011	0.301	0.121	0.257	0.698
	Diurnal mean	0.295	0.080	0.785	0.002	0.066	0.273
R_p/A_{gross}	8-9:00 h	0.597	0.003	0.366	0.896	0.005	0.304
	10-11:00 h	0.846	0.001	0.719	0.007	0.001	0.058
	13-14:00 h	0.127	0.089	0.127	0.019	0.034	0.807
	16-17:00 h	0.269	0.108	0.123	0.711	0.008	0.383
	Diurnal mean	0.881	<.0001	0.585	0.007	<.0001	0.068
ETR	8-9:00 h	0.754	0.771	0.378	0.774	0.467	0.063
	10-11:00 h	0.254	0.275	0.878	0.013	0.620	0.979
	13-14:00 h	0.683	0.095	0.828	0.500	0.500	0.516
	16-17:00 h	0.054	0.802	0.323	0.597	0.692	0.744
	Diurnal mean	0.992	0.461	0.891	0.371	0.711	0.145
qP	8-9:00 h	0.890	0.623	0.859	0.875	0.185	0.343
	10-11:00 h	0.775	0.228	0.827	0.649	0.755	0.310
	13-14:00 h	0.580	0.060	0.660	0.384	0.187	0.489
	16-17:00 h	0.032	0.426	0.276	0.398	0.738	0.683
	Diurnal mean	0.373	0.198	0.662	0.962	0.109	0.109

F_v'/F_m'	8-9:00 h	0.420	0.185	0.060	0.441	0.181	0.670
	10-11:00 h	0.031	0.613	0.613	0.613	0.364	0.157
	13-14:00 h	0.152	0.833	0.674	0.891	0.147	0.891
	16-17:00 h	0.849	0.174	0.899	0.548	0.968	0.968
	Diurnal mean	0.145	0.363	0.273	0.327	0.119	0.638
LS	growth CO_2	0.350	0.005	0.004	0.566	0.068	0.986
LM		0.000	<.0001	<.0001	0.763	0.002	0.861
LB		0.376	0.250	0.157	0.250	0.173	0.924
Glucose	6:00 h	0.697	0.013	0.108	-	-	-
	12:00 h	0.956	0.017	0.418	0.590	0.838	0.688
	18:00 h	0.816	0.047	0.381	-	-	-
Fructose	6:00 h	0.470	0.497	0.396	-	-	-
	12:00 h	0.035	0.017	0.260	0.136	0.096	0.148
	18:00 h	0.382	0.302	0.548	-	-	-
Sucrose	6:00 h	0.053	0.016	0.033	-	-	-
	12:00 h	0.132	0.010	0.542	0.917	0.997	0.074
	18:00 h	0.026	0.172	0.682	-	-	-
Starch	6:00 h	0.542	0.018	0.059	-	-	-
	12:00 h	0.845	0.088	0.016	0.577	0.001	0.619
	18:00 h	0.617	0.037	0.085	-	-	-
Malate	6:00 h	0.023	0.028	0.147	-	-	-
	12:00 h	0.237	0.001	0.266	0.405	0.012	0.151
	18:00 h	0.017	0.014	0.748	-	-	-
g_{m_EDO}		0.627	0.049	0.636	0.835	0.467	0.447
g_{m_Harley}		0.324	0.080	0.551	0.277	0.090	0.742
V_{cmax} (C_c basis)		0.261	0.100	0.626	0.715	0.477	0.356
J_{max} (C_c basis)		0.467	0.052	0.488	-	-	-
C_c at growth CO_2		0.042	0.013	0.375	0.586	0.010	0.688
C_{c_trans}		0.166	0.001	0.165	-	-	-
V_{cmax} (C_i basis)		0.748	0.341	0.261	0.210	0.102	0.867
J_{max} (C_i basis)		0.803	0.639	0.928	-	-	-
C_i at growth CO_2		0.551	<.0001	0.053	0.117	0.022	0.440
C_{i_trans}		0.492	0.742	0.128	-	-	-
LS	Different from growth CO_2	0.228	0.008	0.001	0.728	0.003	0.418
LM		0.000	<.0001	<.0001	0.541	<.0001	0.246
LB		0.541	0.873	0.026	0.190	0.250	0.735

Supplementary Table S2. The effect of elevated (550 µmol mol^{-1}) or ambient (390 µmol mol^{-1}) [CO_2] on some photosynthetic parameters of two coffee cultivars (Catuaí and Obatã) growing in a FACE trial during the growing season (February) and winter (August): the maximum apparent carboxylation capacity (V_{cmax}) and the *in vivo* maximum rate of carboxylation as limited by electron transport (J_{max}), both on a intercellular [CO_2] basis; and the internal [CO_2] (C_i) and C_i of transition (C_{i_trans}). Data for J_{max} and C_{i_trans} were not obtained in August. V_{cmax} and J_{max} were normalised to 25°C using the temperature response equations from Sharkey *et al.* (2007). n = 5-6 ± SE

Parameters (C_i basis)	Catuaí			
	February		August	
	390 µmol mol^{-1}	550 µmol mol^{-1}	390 µmol mol^{-1}	550 µmol mol^{-1}
V_{cmax}	39.2 ± 1.9	37.0 ± 4.7	29.2 ± 1.3	35.1 ± 4.5
J_{max}	73.8 ± 11.4	68.9 ± 7.8	-	-
C_i	248 ± 11	345 ± 15	194 ± 13	260 ± 18
C_{i_trans}	390 ± 19	371 ± 18	-	-
	Obatã			
	February		August	
	390 µmol mol^{-1}	550 µmol mol^{-1}	390 µmol mol^{-1}	550 µmol mol^{-1}
V_{cmax}	41.3 ± 2.9	35.8 ± 2.9	25.6 ± 3.5	30.5 ± 1.9
J_{max}	70.4 ± 12.4	67.1 ± 8.2	-	-
C_i	225 ± 14	387 ± 21	177 ± 17	211 ± 34
C_{i_trans}	355 ± 12	385 ± 12	-	-

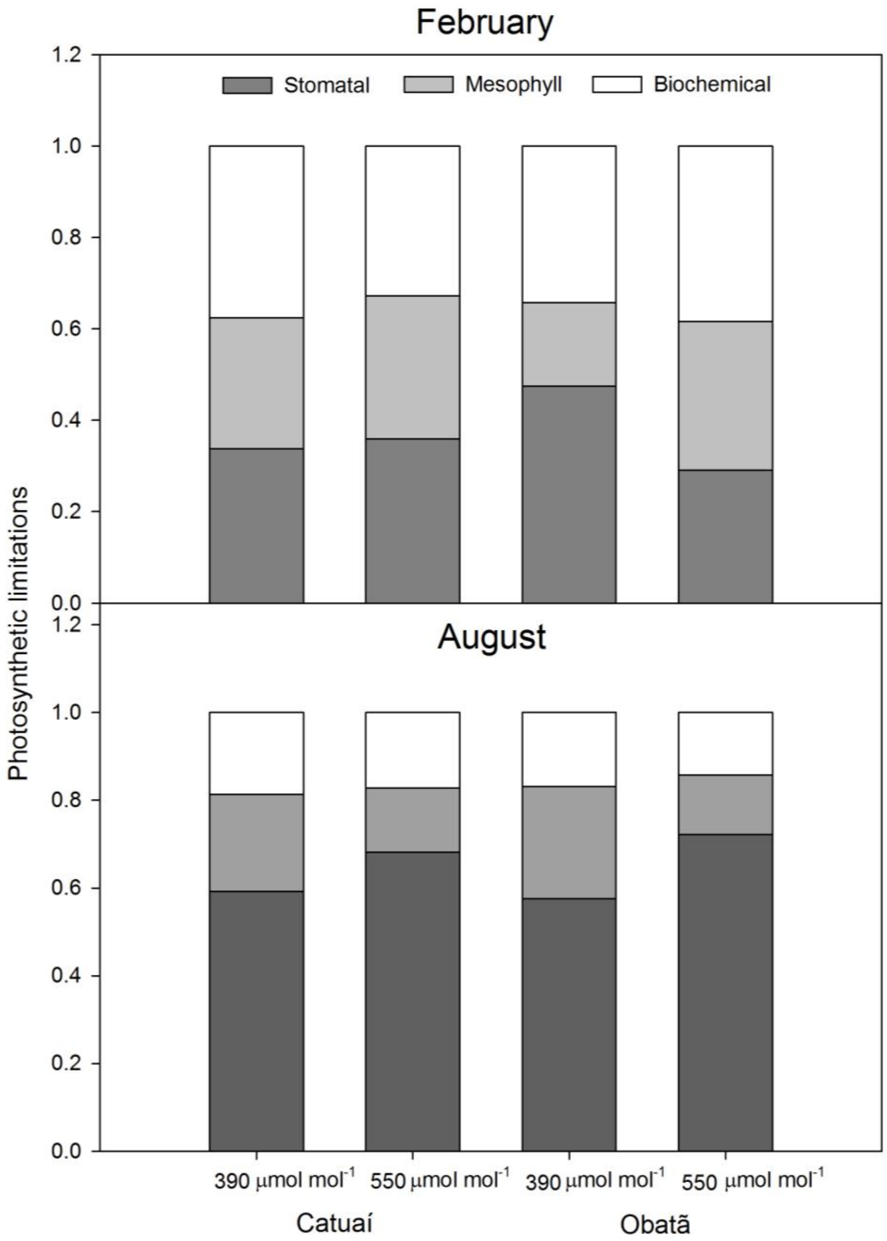

Supplementary Figure S2. The effect of elevated (550 µmol mol^{-1}) or ambient (390 µmol mol^{-1}) [CO_2] on the overall limitations to photosynthesis of two coffee cultivars (Catuaí and Obatã) growing in a FACE trial during the growing season (February) and winter (August): stomatal (l_s), mesophyll (l_m) and biochemical (l_b) limitations. For these estimations, different ambient [CO_2] were used: 390 µmol mol^{-1} air for the plants grown at 550 µmol mol^{-1} air and vice-versa. n = 5-6 ± SE
